# Hospital staff education on severe sepsis/septic shock and hospital mortality: an original hypothesis

**DOI:** 10.1186/1471-2253-12-28

**Published:** 2012-11-20

**Authors:** Maurizia Capuzzo, Marco Rambaldi, Giovanni Pinelli, Manuela Campesato, Antonia Pigna, Marco Zanello, Maria Barbagallo, Massimo Girardis, Elena Toschi

**Affiliations:** 1 Azienda Ospedaliero-Universitaria S. Anna of Ferrara, Department of Emergency, University Unit of Anaesthesia and Intensive Care, Via Aldo Moro 8 Cona, Ferrara, 44124, Italy; 2 Azienda Unità Sanitaria Locale of Modena, Hospital “Baggiovara”, Unit of Emergency Medicine, Modena, Italy; 3 Azienda Ospedaliero-Universitaria of Bologna, Department of Emergency/Urgency, General and Transplant Surgery, Unit of Anaesthesiology and Intensive Care “Professor Faenza”, Bologna, Italy; 4 Azienda Ospedaliero-Universitaria of Bologna, Department of Specialized Surgeries and Anaesthesiology, Unit of Anaesthesiology “Professor Di Nino”, Bologna, Italy; 5 Istituto di Ricovero e Cura a Carattere Scientifico delle Scienze Neurologiche “Bellaria” of Bologna, Unit of Anaesthesia and Intensive Care, Bologna, Italy; 6 Azienda Ospedaliero-Universitaria of Parma, Department of Surgery, Unit 2nd of Anaesthesia, Intensive Care and Pain therapy, Parma, Italy; 7 Azienda Ospedaliero-Universitaria of Modena, Department of General and Specialized Surgeries, Unit I of Anaesthesia and Intensive Care, Modena, Italy

## Abstract

**Background:**

Signs of serious clinical events overlap with those of sepsis. We hypothesised that any education on severe sepsis/septic shock may affect the outcome of all hospital patients. We designed this study to assess the trend of the mortality rate of adults admitted to hospital for at least one night in relationship with a hospital staff educational program dedicated to severe sepsis/septic shock.

**Methods:**

This study was performed in six Italian hospitals in the same region. Multidisciplinary Sepsis Teams members were selected by each hospital management among senior staff. The education included the following steps: i) the Teams were taught about adult learning, problem based learning, and Surviving Sepsis guidelines, and provided with educational material (literature, electronic presentations, scenarios of clinical cases for training and booklets); ii) they started delivering courses and seminars each to their own hospital staff in the last quarter of 2007.

To analyse mortality, we selected adult patients, admitted for at least one night to the wards or units present in all the study hospitals and responsible for 80% of hospital deaths. We fitted a Poisson model with monthly hospital mortality rates from December 2003 to August 2009 as dependent variable. The effect of the educational program on hospital mortality was measured as two dummy variables identifying a first (November 2007 to December 2008) and a second (January to August 2009) education period. The analysis was adjusted for a linear time trend, seasonality and monthly average values of age, Charlson score, length of stay in hospital and urgent/non-urgent admission.

**Results:**

The hospital staff educated reached 30.6% at the end of June 2009. In comparison with the pre-education period, the Relative Risk of death of the patient population considered was 0.93 (95% confidence interval [CI] 0.87-0.99; p 0.025) for in-patients in the first, and 0.89 (95% CI 0.81-0.98; p 0.012) for those in the second period after education.

**Conclusion:**

Our hypothesis that a program educating hospital staff to early detection and treatment of severe sepsis/septic shock may affect the outcome of all hospital patients is original, but it has to be corroborated by other experiences.

## Background

Severe sepsis is frequent, responsible for high mortality and costly [1,2]. Hospital mortality ranges from 20.7% to 55.2% for severe sepsis and 40.9% to 60.5% for septic shock [3-8]. To face the tremendous burdens of this problem, the Surviving Sepsis Campaign guidelines were developed, published in 2004 and updated in 2008 [9,10].

The clinical signs of sepsis [11] are quite unspecific and require the observation of basic vital signs like body temperature, heart rate, respiratory rate, consciousness, blood pressure, and urine output. Laboratory tests are necessary but clinical observation of the patient, either septic or non septic, is the prerequisite for identifying patients at risk of any serious clinical events, frequently preceded by an antecedent sign of clinical deterioration [12]. Interestingly, early intervention in the care of critically ill patients is strongly beneficial [13] . However, it requires early detection of the patient’s clinical deterioration by the staff, who are frequently unaware of their patients’ abnormal vital signs [14].

Since signs of clinical deterioration in ward patients overlap with signs of severe sepsis and septic shock (SS/SS), we hypothesised that any education dedicated to early recognition and treatment of signs of SS/SS may affect the outcome of all hospital patients. Therefore, we designed a study to assess the trend of the mortality rate of adults admitted to hospital for at least one night in relationship with a hospital staff educational program on severe sepsis/septic shock.

## Methods

This study was performed in four University and two community hospitals in the Italian region Emilia-Romagna. The number of hospital beds ranges from 740 to 1308 for University hospitals, and 385 to 484 for Community hospitals. The clinical staff, including physicians and nurses and excluding staff involved in administrative duties, ranged from 1546 to 2862 for the former, and 610 to 865 for the latter on the 31 December 2008. Each study hospital had an Emergency Department.

The wards of these hospitals have at least one senior attending physician, fellow physicians taking care of 6–8 patients each, and residents during office hours (08.00-20.00). A senior attending physician is always present at night and during weekends. The nurses check and record vital signs of the patients at least three times a day (usually at 8.00, 16.00 and 24.00). The senior and fellow physicians prescribe diagnostic tests and treatments and they are the reference physician for the ward nursing staff. Whenever they are worried about a patient they call for the intensivist consultation, defining at the time of the call one of the following degrees of urgency: emergency, that is immediate, or urgent, within maximum 30 minutes, or non urgent (within max 6h).

At the 31 December 2008, the number of adult Intensive Care Unit (ICU) beds per hospital ranged from 12 to 60. The Intensive Care Units of these hospitals are closed with at least one physician specialist in intensive medicine present 24/24 and 7/7, and a nurse to patient ratio of 1:2. The study hospitals do not have a formally dedicated Rapid Response Team and calls from wards or Emergency Department are managed by the ICU medical staff.

### Educational program

In 2006, the Emilia-Romagna Regional Health Agency launched the project “Lotta alla Sepsi in Emilia-Romagna” (fight against sepsis, acronym LaSER) with the objective of educating hospital staff in the early detection and effective treatment of SS/SS, according to the Surviving Sepsis campaign [9,10]. The specific aim of this project was to transfer to the clinical practice the following interventions: early recognition of the septic patient (focusing on clinical signs of sepsis, inflammatory and organ dysfunction variables), early initial resuscitation (fluid and vasoactive amine administration, and goals), microbiological diagnosis (appropriate cultures, source identification and control when required), and early antibiotic treatment (empiric, intravenous with de-escalation).

**Figure 1:**
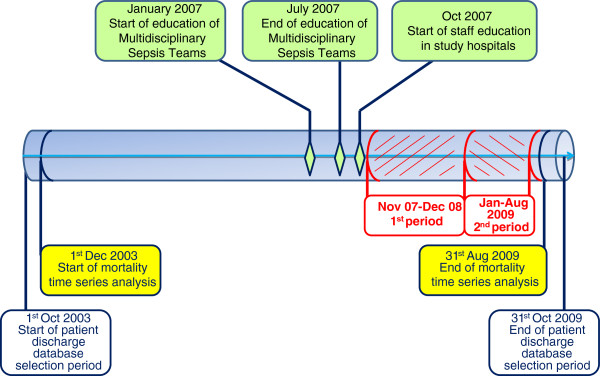
Timeline of the study activities.

The Regional Health Agency arranged an educational package consisting of information about epidemiology, morbidity and mortality of SS/SS, scientific literature, electronic presentations for lectures, format of clinical cases for practice training, and booklets reporting clinic and laboratory signs of SS/SS [15]. In the first three quarters of 2007 (Figure 1), the Regional Health Agency acted as coordinating centre and organized meetings with the Multidisciplinary Sepsis Teams. The Multidisciplinary Sepsis Teams included doctors and nurses of infectious disease wards, intensive care, and the emergency department, a microbiologist and a pharmacist, selected by each hospital management among the senior staff. They participated in a course where they were taught about principles of adult learning [16], problem based learning [17] and Surviving Sepsis guidelines. The Multidisciplinary Sepsis Teams were provided with educational material (scientific literature, electronic presentations for lectures, scenarios of clinical cases for practice training and booklets) and started delivering courses and seminars each to their own hospital staff in the last quarter of 2007 (Figure 1). The methodology adopted for the educational courses in each hospital included delivery of short lectures, discussions as well as problem based learning on SS/SS scenarios. A typical course session held in the study hospital lasted four hours (14:30 to 18:30), included the presentation of the objectives of the course, definition, general and local epidemiology, early recognition, early goal-directed therapy, microbiological diagnosis, and early antibiotic treatment of SS/SS.

The target of the present educational program consisted of attending physicians and nurses involved in direct patient care, excluding staff involved in only administrative duties. Residents were free to attend the educational sessions but they were not included either in the clinical staff or in the educated staff. The clinicians who are authors of the present study were strongly involved in the education project in their hospitals. The education involved firstly the larger wards, intensive care and emergency department in all hospitals, with lectures and clinical cases presented to nurses and physicians together. Participation in the courses was free and voluntary, but credits for continuing medical education were given to the attendees.

### Data source of general hospital mortality

The study country has a National Health Service, organized into Regional Health Agencies. Since 2000, diagnosis and procedures have been codified according to the International Classification of Diseases, Ninth Revision, Clinical Modification (ICD-9-CM) codes. Hospital data on admissions are collected in the (*Scheda di Dimissione Ospedaliera - SDO*) SDO discharge database. We conducted the analysis of hospital mortality on the (Scheda di Dimissione Ospedaliera) SDO discharge database for the Emilia-Romagna region, containing personal anonymous data and hospitalisation data.

We selected from the discharge SDO database the records meeting the following criteria: i) admission in the period from October 2003 to October 2009; ii) admission with discharge on a different day; iii) age ≥16 years; iv) admissions not resulting in transfer to another acute hospital; v) admitted to the wards/units of the nine specialties reporting at least 130 deaths per year. This number corresponds to the wards which have been responsible for 81.7% of the cumulative hospital mortality in the period from October 2003 to October 2009. This last selection was performed in order to highlight the effect of education in the wards most involved in the outcome of interest (hospital mortality). For each selected admission we calculated the Charlson score using the Charlson Comorbidity Index SAS macro code [18].

The following Ethics Committees approved the study and waived informed consent: Comitato Etico Provinciale di Ferrara (in the meeting held on 25^th^ Oct 2007), Comitato Etico Provinciale di Modena (Protocol N. 94/09), Comitato Etico Indipendente di Azienda Ospedaliero-Universitaria di Bologna (Protocol N. CE 40/2008/U/Oss), Comitato Etico Indipendente di Azienda Unità Sanitaria Locale di Bologna (Protocol N. 1311/CE), and Comitato Etico Unico per la Provincia di Parma (Protocol N. 25997).

#### Statistical analysis

Categorical data are reported as number with percentages, and continuous variables as mean ± 1 SD. F-test (one-way ANOVA) was used to compare means and Chi-Squared test to compare percentages of categorical variables. Statistical analysis was performed using the software R version 2.11.0 [19].

We performed a time series analysis, modelling a sequence of data points (dependent variable) measured at successive time instants, spaced at uniform time intervals. Admission data were available from October 2003 to October 2009. We used months as time intervals and the dependent variable was the monthly hospital mortality rate calculated for each of the 69 months from December 2003 to August 2009. We excluded data regarding the two months before this period in order to include all the patients present in hospital every month. For each month we computed the mortality rate for the patients present in hospital in that month. We also excluded data regarding the last two months because our data came from a retrospective discharge dataset, so data regarding the last two months could be incomplete and missing the patients admitted in that period and not yet discharged. The SDO records are collected on an individual patient basis, so we collapsed data into a time series of monthly average values or proportions in order to model the mortality rate trend over time (months). A Poisson regression model which is suitable for any situation that involves counting events was developed. We expected that changes in patient characteristics and treatments had occurred over more than six years. Therefore the analysis was adjusted for a linear time trend (which takes into account each month and sums up all those modifications that may have occurred over the previous time), seasonality (11 monthly indicator variables) and monthly average values of four variables which had a potentially confounding effect (age, Charlson score, hospital length of stay and urgent/non-urgent admission). The effect of the education on hospital mortality was measured as the coefficients of two dummy variables identifying a first (November 2007 to December 2008) and a second education period (January to August 2009). Durbin-Watson statistics and visual inspection of the residuals versus time were used to check for possible autocorrelation.

## Results

The study timeline with milestones is reported in Figure 1. The training of the staff involved firstly larger wards in all the study hospitals and started in the last quarter of 2007. The cumulative percentage of hospital staff educated was 25.2% at the end of December 2008 and reached 30.6% at the end of June 2009. The hospital and staff characteristics, and the numbers of staff members who attended the educational program are reported in Table 1. The percentage of educated staff was lower in the larger hospitals (B and D).

**Table 1:** Characteristics of the study hospitals

**Hospital**	**A**	**B**	**C**	**D**	**E**	**F**	**Total**
N. of hospital beds	742	1308	740	1231	345	484	4850
N. of ICU beds for adults	16	60	19	37	12	20	164
% of beds devoted to intensive care	2.2	4.6	2.6	3.0	3.5	4.1	3.4
N. of physicians	470	867	494	607	177	243	2858
N. of nurses	1076	1995	1064	1657	433	622	6847
N. of hospital staff	1546	2862	1558	2264	610	865	9705
**Staff who attended educational program (%)**							
N. of physicians at 31 December 2008	137 (29.1)	125 (14.4)	200 (40.5)	108 (17.8)	37 (10.7)	67 (13.8)	674
N. of physicians at 30 June 2009	165 (35.1)	152 (17.5)	230 (46.6)	128 (21.1)	53 (15.4)	72 (14.9)	800
N. of nurses at 31 December 2008	296 (27.5)	337 (16.9)	418 (39.3)	351 (21.2)	157 (36.3)	208 (33.4)	1767
N. of nurses at 30 June 2009	347 (32.2)	437 (21.9)	496 (46.6)	471 (28.4)	172 (39.7)	246 (39.5)	2169
**Percentages of hospital staff educated**							
at 31 December 2008	28.0	16.1	39.7	20.3	31.8	31.8	25.2
at 30 June 2009	33.1	20.6	46.6	26.5	36.9	36.8	30.6

Physician and nursing staff directly involved in patient care present in each hospital at 31 December 2008.

There were 411,543 overnight adult records of admission selected from the SDO database according to the criteria illustrated above. The following nine specialities were included in the analysis: Internal medicine, Geriatrics, Emergency Department, Pneumology, Intensive Care, Oncology, General Surgery, Coronary Care Unit, and Infectious Disease. The characteristics of the patients included in the mortality analysis before hospital staff education, in the first and in the second period after starting hospital staff education are reported in Table 2. Age, Charlson index, hospital deaths and percentages of females and of urgent admissions were lower in the period before education than in the other periods.

**Table 2:** Characteristics of the patients included in the mortality analysis before hospital staff education, in the first and in the second period after starting hospital staff education

**Period**	**Before education**	**First period of education**	**Second period of education**	**Total**	**p-value**
Patients	276,541	78,052	56,950	411,543	
Age (years)	67.6 ± 17.3	68.5 ± 17.3	68.5 ± 17.3	67.9 ± 17.3	< 0.0001
Gender male (%)	139,350 (50.3)	39,063 (50.0)	28,376 (49.8)	206,789 (50.2)	0.0229
Charlson index	4.7 ± 1.8	4.8 ± 1.9	4.9 ± 1.9	4.7 ± 1.8	< 0.0001
Urgent admission (%)	208,660 (75.4)	59,088 (75.7)	43,241 (75.9)	310,989 (75.5)	0.0345
LOS (days)	11.0 ± 14.2	11.2 ± 13.8	10.6 ± 12.0	11.0 ± 13.8	< 0.0001
Hospital deaths (%)	17,270 (6.2)	5,237 (6.7)	3,732 (6.6)	26,239 (6.4)	< 0.0001

Values are reported as number with percentage, or mean ± SD.

*LOS*: Length Of Stay in hospital.

**Figure 2:**
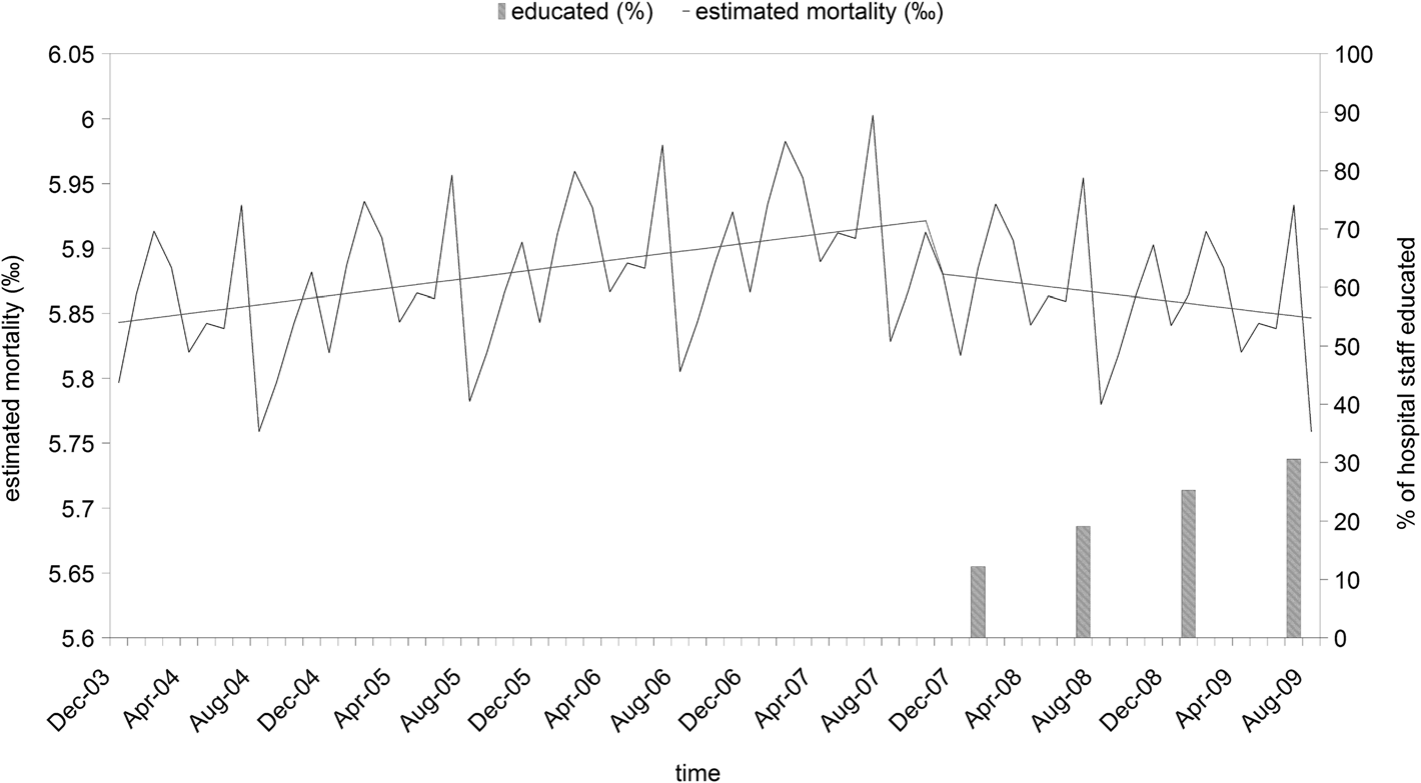
**Estimated monthly hospital mortality rates for a fixed number of average patients (line) and percentages of educated staff (as bars).** The plot shows the estimated monthly hospital mortality rate for a fixed number of patients with mean values of age, Charlson index, hospital length of stay and mean percentage of urgent hospital admission. The analysis was performed on a period of 69 months (from December 2003 to August 2009). The education started in the last quarter of 2007.

The adjusted monthly hospital mortality rates for a fixed number of average patients are reported in Figure 2, where the cumulative percentages of hospital staff educated are shown on a six-month basis. The two dummy variables identifying first (November 2007 to December 2008) and second (January to August 2009) period of education where found to be statistically significant in the Poisson model. Their coefficients were −0.072 and −0.116, respectively. In comparison with the period before education (December 2003 to October 2007), the Relative Risk (RR) of death for the in-patients in the period November 2007 to December 2008 was 0.93 (95% Confidence Interval [CI] 0.87-0.99; p 0.0251), and the RR for the in-patients in the period from January to August 2009 was 0.89 (95% CI 0.81-0.98; p 0.0128).

## Discussion

The positive effect of SS/SS education on care and outcome of ICU patients with SS/SS has been already demonstrated [20,21]. In these as in our study, efforts to improve the care of SS/SS patients targeted nearly all physicians specialties, because a wide variety of medical specialties care for severe sepsis patients [22]. Nevertheless, clear information about the number of staff educated is generally lacking in the literature. Ferrer et al. [20] standardized the educational program and used multidisciplinary teams to deliver education to the staff of emergency department, medical and surgical wards, and ICUs. Their before-after study demonstrated a significant reduction in hospital mortality, but they studied only the patients with SS/SS admitted to 59 ICUs and reported a mean time dedicated to lectures of 10.6 hours at each center, without mentioning the number of the trained staff members. In a study performed in 165 hospitals, Levy et al. [23] found that a multi-faced intervention to facilitate compliance with the guidelines of the Surviving Sepsis Campaign in ICU, emergency department and wards was associated with a decrease of hospital mortality of the severe sepsis patients enrolled in an *ad hoc* database. No information was given about the number of the trained staff or the time dedicated to education [23]. At hospital level, a multi-format educational approach on sepsis for all workers involved in patient care in a single institution was effective in reducing the mortality of the patients with sepsis [24]. Moreover, a recent multicenter study using both before-and-after and concurrent analyses assessed the in-hospital mortality effect of "GENeralized Early Sepsis Intervention Strategies" in community and tertiary care settings [25]. The patients with severe sepsis and septic shock receiving the 6-h resuscitation bundle via a sepsis team or sepsis order sets experienced a significant reduction in mortality [25]. On the other hand, the training of 50% of the doctors and 70% of the nurses of four wards by medical simulation in a single hospital [26] did not increase the staff awareness of the patients at risk despite a similar incidence of patients with abnormal vital signs in the pre and post-intervention periods [27]. The reason for this result may relay on the lack of routine assessment of vital signs for all ward patients in the study hospital, as reported by the authors in a previous study [14], and not changed in the meanwhile [27].

Our educational project on SS/SS involved only 30% of the hospital clinical staff, but other unmeasured factors may have influenced the number of educated staff in the wards and units involved in the analysis of mortality. Firstly, the education started involving the staff of emergency departments, ICUs and larger wards, which were also those included in the study and with higher mortality. The lower percentages of educated staff found in larger hospitals should be due to the lack of education of the staffing for specialised wards with very low mortality (for example allergology, endocrinology, rheumatology), because they were not involved in this phase of the educational project. As a consequence, the percentage of the educated hospital staff is certainly underestimated for the wards/units included in the present analysis. Secondly, some spread of the Surviving Sepsis Campaign guidelines happened before starting the present project and was strengthened by the publication of the updated version of the Surviving Sepsis Campaign [10]. We don't have any measure of this spread but our time series analysis started long before. Moreover, the Multidisciplinary Sepsis Teams consisted of senior staff who acted as tutors for the problem based learning used in the education of our target staff, and this may have facilitated the learning of our well experienced (we excluded residents and medical students) hospital staff [28].

We found changes in patient characteristics over the long time of the study. As expected, patients' age and incidence of female gender increased as a consequence of the increased life expectancy, especially in females. Comorbidity index and urgent admissions also increased, while length of stay in hospital decreased. However, the time series analysis took into account all those modifications to give a result purified of these slow changes. We did not distinguish the populations with and without SS/SS due to the following reasons: i) our data were collected for administrative reasons (reimbursement) and diagnosis codes may not be properly recorded by the individual physician [29]; ii) it has not been definitely clarified which international classification of diseases codes correspond to the definition of severe sepsis accepted by the ACCP/SCCM; iii) the coexistence of diagnostic codes for infection and organ failure does not necessarily mean that a causal or temporal relation exists [30]. Finally, we can speculate that international classification of diseases coding habits may be influenced by culture, and training courses aiming at improving attention to a clinical phenomenon can sensitize health professionals in the detection of that clinical phenomenon and increase their reporting.

Our original never tested hypothesis is that teaching hospital staff to early recognize and treat SS/SS should allow early detection of any patient deterioration, regardless of the presence of sepsis. The consequent early medical intervention (like early goal-directed therapy in septic patients [31]) on ward patients at risk may allow clinical improvement, prevent further deterioration, and finally reduce the hospital mortality. A recently published study on retrospective cardiac arrest registry data has compared in-hospital cardiac arrests occurred within 72 h of hospital admission in patients with (4,453) and without (32,645) preexisting pneumonia [32]. Only 36.5% of the patients with and 30% of those without preexisting pneumonia were receiving mechanical ventilation and only 33.3% of the patients with and 28.8% of those without preexisting pneumonia were receiving infusions of vasoactive drugs prior to cardiac arrest. These results show that more than half of the cardiac arrests occurred in the lack of overt shock and respiratory failure, suggesting the need of early recognition of clinical deterioration in patients with and without sepsis [32]. Moreover, it has long been shown that, in comparison with the patients developing septic shock in the ICU, those developing septic shock in general ward receive intravenous fluid boluses and vasoactive agents with clinically important delay [33]. Unfortunately we don't know whether our educated staff may have early identified or earlier and/or better treated clinical deterioration, or both according to the education received. Nevertheless sufficient evidence of adverse events like hospital mortality and ICU admission already exists; there is a need now for strategies to prevent or reduce them [34]. We could also hypothesise that our finding was due to the ability of the educated staff to assess and/or treat only the patients with SS/SS. The incidence of severe sepsis in 2006 found by the Regional Health Agency using the algorithms suggested in the literature [1] ranged from 1.08 to 3.36 per 1000 hospital admissions (unpublished data). Even doubling this incidence it does not appear reasonable to hypothesize that the effect found in this study was due to the education directed to such a small number of patients having SS/SS. Moreover, it seems unlikely, but we cannot exclude either an undetected change in the case-mix of hospital patients (the epidemic peak of A(N1H1) influenza in Italy was after the period of the present study [35]), or an Hawthorne effect.

This study has strengths and weaknesses. Our study is limited by its observational design, which cannot exclude the possibility of our results being confounded by case-mix heterogeneity, but appropriately designed observational studies can provide valuable information on intervention effect [36]. We assessed the percentages of staff educated in the study hospitals, but not those of the staff working in the wards or units assessed for mortality or those previously performed by the staff. We were able to identify only the starting time of the educational program, because the Regional Health Agency opted to continue education. Finally, we did not address specifically the patients with SS/SS.

There are other weaknesses of the study. Firstly, limited information to characterize the population was available, so the hospital mortality was adjusted using only four patient-related variables, also if the linear time trend took into account for each month all modifications occurred over the previous time and seasonality. Secondly, we don't have any data about the compliance with treatment guidelines or any quality indicators assessing the change in processes of care as training result. Thirdly, someone could argue that the long period considered in the time series analysis could have compromise the ability to associate the reduction in mortality with education, but the first version of the Surviving Sepsis Campaign was published in March 2004, so we expected some effect of the natural spread of the Campaign. Finally, 30% of trained staff may be a low target to associate training with mortality reduction, but results of education may depend not only on quantity but also on quality of education, as well as on other factors difficult to measure like educational activities individually performed by staff members and leadership of tutors.

Our hypothesis that a program educating hospital staff to early detection and treatment of severe sepsis/septic shock may affect the outcome of all hospital patients is original, but it has to be corroborated by other experiences. We hope that investigators who manage wide databases in countries strongly involved in Surviving Sepsis Campaign will test our hypothesis.

## Conclusions

This study suggests that an educational program specifically devoted to severe sepsis/septic shock according to the Surviving Sepsis Campaign was associated with a decrease in the hospital mortality of the patients admitted to the hospital wards/units responsible for most of the cumulative hospital mortality. If this finding is confirmed, the information would be useful for hospital administrations and health policy makers.

## Abbreviations

LOS: Length of stay; RR: Relative risk; SDO: (Scheda di Dimissione Ospedaliera) discharge database; SS/SS: Severe sepsis and septic shock. Some preliminary data were presented at the 22^nd^ Annual Congress of the European Society of Intensive Care Medicine held in Vienna (11–14 Oct 2009) and as an abstract at the congress “Ricerca & innovazione nel servizio sanitario dell’Emilia-Romagna” (Bologna, 18–19 Jan 2010).

## Competing interests

The authors declare that they do not have any potential conflict of interests related to the present manuscript.

## Authors’ contributions

Dr Capuzzo had full access to all the data in the study and takes responsibility for the integrity of the data and the accuracy of the data analysis. MC, GP, MZ and MG conceived the study. ET performed data extraction and statistical analysis, MR, MCampesato, AP, MZ, MB, and MG are clinical investigators who take care of the educational project in the study hospitals and critically revised the draft manuscript. All the authors approved the final submission.

## Pre-publication history

The pre-publication history for this paper can be accessed here:

http://www.biomedcentral.com/1471-2253/12/28/prepub

## Aknowledgements

The authors thank Prof Alberto Barbieri and Dr Paolo Piccinini (Azienda Ospedaliero-Universitaria of Modena), and Dr Elisabetta Spadini (Azienda Ospedaliero-Universitaria of Parma) for their help in data education, and Dr Maria Luisa Moro and Dr Davide Resi (Agenzia Sociale Sanitaria Regione Emilia-Romagna) for organizing the LaSER campaign. The authors also thank the following physicians who worked as site researchers: Alberto Andreotti, Roberta Benini, Elena Bergamini, Angela Berselli, Susanna Biondini, Caterina Curti, Giuseppe Loschi, Alessandra Markidis, Carlotta Moretti, and Raffaella Papa.
